# HER3 Differentiates Basal From Claudin Type Triple Negative Breast Cancer and Contributes to Drug and Microenvironmental Induced Resistance

**DOI:** 10.3389/fonc.2020.554704

**Published:** 2020-11-20

**Authors:** Nicoleta Sinevici, Bahar Ataeinia, Veronica Zehnder, Kevin Lin, Lauren Grove, Pedram Heidari, Umar Mahmood

**Affiliations:** Department of Radiology, Massachusetts General Hospital and Harvard Medical School, Boston, MA, United States

**Keywords:** triple negative breast cancer, resistance, tyrosine kinase inhibitors, microenvironment, neuregulin

## Abstract

Triple Negative Breast Cancer (TNBC) is an aggressive form of Breast Cancer (BC). Numerous kinase inhibitors (KI) targeting different pathway nodes have shown limited benefit in the clinical setting. In this study, we aim to characterize the extent of HER3 reliance and to define the effect of Neuregulin (NRG) isoforms in TNBCs. Basal and Claudin type TNBC cell lines were treated with a range of small molecule inhibitors, in the presence or absence of the HER3 ligand NRG. Single agent and combination therapy was also evaluated in human cancer cell lines through viability and biochemical assessment of the AKT/MAPK signaling pathway. We show that Basal (BT20, HCC-70, and MDA-MB-468) and Claudin type (MDA-MB-231, BT-549) TNBC cell lines displayed differential reliance on the HER family of receptors. Expression and dynamic HER3 upregulation was predominant in the Basal TNBC subtype. Furthermore, the presence of the natural ligand NRG showed potent signaling through the HER3-AKT pathway, significantly diminishing the efficacy of the AKT and PI3K inhibitors tested. We report that NRG augments the HER3 feedback mechanism for continued cell survival in TNBC. We demonstrate that combination strategies to effectively block the EGFR-HER3-AKT pathway are necessary to overcome compensatory mechanisms to NRG dependent and independent resistance mechanisms. Our findings suggests that the EGFR-HER3 heterodimer forms a major signaling hub and is a key player in tumorigenesis in Basal but not Claudin type TNBC tested. Thus, HER3 could potentially serve as a biomarker for identifying patients in which targeted therapy against the EGFR-HER3-AKT axis would be most valuable.

## Introduction

Triple Negative Breast Cancer (TNBC) is an aggressive, heterogenous disease in which targeted therapy has shown limited efficacy. TNBC affects approximately 15-20% of breast cancer (BC) patients, usually of reproductive age, and shows the highest rate of recurrence and mortality within the BC subgroups (1). TNBC is characterized by the lack of expression of Estrogen Receptor (ER), Progesterone Receptor (PR) or the HER2 receptor (2). Lack of targetable therapy options in TNBC significantly limits the therapeutic arsenal and to date the standard of care remains chemotherapy (3). Numerous kinase inhibitors (KI) targeting different pathway nodes including the PI3K, AKT and MAPK pathway have shown limited benefit in the clinical setting (4). Intrinsic and extrinsic resistance mechanisms are thought to be the main reason of therapeutic failure, including mechanisms related to signaling through members of the Epidermal Growth Factor Receptor (EGFR) family.

The EGFR family is arguably one of the most targetable family of receptors in cancer and the overexpression of HER2 in HER2+ BC has dramatically changed the survival outcome of women with this subtype. The interplay and redundancy of the EGF receptors makes single receptor therapeutic intervention rarely curative as feedback mechanisms are quickly able to restore signaling through alternative receptors (5). One such receptor is the HER3 receptor which has gained attention in HER2+ BC as a major player involved in resistance (6). The heterodimeric complex formed between HER2 and HER3 is a particularly potent oncogenic signaling unit and driver of cancer growth. Furthermore, HER3 upregulation in response to small molecule inhibitors has been shown to form a positive feedback mechanism, and has been shown to rescue a large number of cancers including TNBC (7–10). Clinically, the importance of EGFR and HER3 has been confirmed by studies showing up to 76% expression of EGFR and 41% expression of HER3 in TNBC (11, 12).

Although, homo/heterodimerization in the absence of a ligand is possible, engagement relies on spatial and temporal expression of the receptors. Overexpression of the receptor(s) facilitates non ligand dependent mechanisms. Lessons from therapeutic failure in HER2+ BC have demonstrated that while ligand-independent HER2 signaling can be successful with Herceptin, the same inhibitory drug remains ineffective in the ligand-dependent signaling scenario. HER3 can be bound by its natural ligand Neuregulin (NRG) of which there are 15 isoforms (13, 14). NRG can induce signaling in autocrine, paracrine and juxtracrine fashion and its numerous transcript variants can induce receptor specific effects including cellular proliferation, stemness, invasion and metastasis but also paradoxically has been shown to induce cellular death and autophagy (15–17). The NRG-HER3 signaling axis has been intensively studied and its central role in mediating resistance has been demonstrated in numerous cancers, particularly those which show high HER2 expression. Furthermore, Kodack et al., (18), demonstrated differential responses to PI3K and AKT inhibitors in the brain microenvironment opposed to the mammary fat pad which was attributed to high levels of the ligand NRG. NRG has been shown to be expressed in numerous tissues including the breast, brain, ovary, prostate, heart, skeletal muscle, lung, liver, kidney, salivary gland, small intestine and spleen (19, 20). Thus, its presence in the microenvironment may play a significant role in the therapeutic response of TNBCs.

Here, we sought to identify the mechanistic basis of resistance to small molecule inhibition in TNBC with an emphasis on the HER3 receptor within different microenvironments and TNBC subtypes. We report hardwired drug induced differences between Basal and Claudin type TNBC. We show that NRG but not Hepatocyte Growth Factor augments the HER3 feedback mechanism for continued survival in TNBC, particularly in the Basal subtype. In contrast, we show that NRG mediated resistance is dependent on the NRG isoform. We further demonstrate that cellular proliferation is maintained through both the PI3K and MAPK pathway and single targeting is inefficient in overcoming resistance. Thus, stratification of patients that show HER3 expression in response to small molecule inhibitors may be used for identifying patients who would respond to dual targeted inhibition, overcoming both ligand dependent and independent signaling.

## Materials and Methods

### Cell Lines

BT-20, MDA-MB-468, HCC-70, MDA-MB-231, and BT-549 cell lines were obtained from ATCC. Cells were maintained in RPMI-1640 medium supplemented with 10% FBS. Cells were cultured in a humidified incubator at 37 °C, 5% CO_2_.

### Cell Viability Assay

Cells were seeded into 96-well plates followed by treatment with individual kinase inhibitors GDC0068 (Selleckchem), GDC0077, Gefitinib (Selleckchem), Neratinib (Selleckchem), Lapatinib (LC Laboratories), Sapitinib (FisherScientific), and the HER3 Ab (R&D Systems), in the presence or absence of NRG-1α or -1β. Vehicle DMSO (1:1,000) was used as control. For drug combination studies, kinase inhibitors, GDC0068, GDC0077 were used at concentration of 1 μM alone, or in combination with lapatinib (200 nM), gefitinib (200 nM) in the presence or absence of NRG isoforms. Cell viability was evaluated by Celltitre Glo luminescence assay after a 4 day treatment and analyzed by % of control cell growth.

### Cell Lysates Preparation and Western Blot Analysis

Cell were lysed in RIPA buffer supplemented with protease and phosphatase inhibitors (Roche). Equal amounts of proteins from cells were separated by 4%–12% SDS-PAGE and were transferred to a PVDF membrane. The blots were blocked for 1 h with 5% Bovine Serum Albumin and then incubated over night with the following primary antibodies: Anti-EGFR (2256, Cell Signaling), Anti-pEGFR (2234, Cell Signaling), anti-HER2 (SC52349, Santa Cruz), anti-pHER2 (2247, Cell Signaling), anti-HER3 (SC81455, Santa Cruz), anti-pHER3 (4791, Cell Signaling), anti-AKT (2920, Cell Signaling), anti-pAKT (4060S, Cell Signaling) anti-P70S6K (49D7, Cell Signaling), anti-pP70S6K (108D2, Cell Signaling), and anti-GAPDH (ab9485, Abcam). Membranes were incubated with their respective secondary antibodies for 1h and analyzed using the enhanced chemiluminescence (ECL) system. Antibody detection and quantification were conducted using the iBright ™ FL1000 (ThermoFisher) and the iBright analysis software.

### Immunofluorescence

Cells were washed in PBS, fixed in 4% paraformaldehyde for 10 min at 37°C and permeabilized with 0.1% Triton X-100 in 1x PBS for 15 min at RT. Cells were blocked overnight with blocking buffer (10% FBS,0.1% BSA and 0.1% Triton X-100 in PBS) followed by primary antibodies (1:200) incubation in blocking buffer for 2h. Cells were washed with 0.1% Triton-X in PBS and incubated with appropriate fluorescent secondary antibodies (1:500) and nuclear staining (DAPI 1 µg/ml) in blocking buffer for 1h. Cells were washed in 0.1% Triton X-100 in PBS prior to imaging. Immunofluorescent images were captured with a 10X objective using a Cytation 5 Cell Imaging Multi- Mode Reader (BioTek Instruments, Inc., Winooski, VT) configured with DAPI, GFP and Texas Red light cubes. The microscope uses a combination of LED light sources in conjunction with band pass filters to provide the appropriate wavelength of light. The DAPI light cube is configured with a 357/44 excitation filter and a 447/60 emission filter; the GFP light cube uses a 470/22 excitation filter and a 510/42 emission filter; while the Red light cube uses a 585/29 excitation and 624/40 emission filters. To compare protein expression levels, images were taken with the same exposure time. Images were captured and stored in TIF format using the Cytation 5 and GEN 3.03 data analysis software (BioTek Instruments, Inc., Winooski, VT).

### Bioinformatic Analysis

DepMap (Broad Institute, https://depmap.org/portal/) was explored for EGFR, HER2, HER3 and NRG1 expression in triple negative breast cancer cell lines based on the Expression Public 20Q2 database and stratified by Basal A and Basal B subtypes.

### Statistical Analysis

The results were obtained from at least three independent experiments and are expressed as means ± standard error of the mean. Data were analyzed with GraphPad Prism statistical software 8.0 (GraphPad Software, La Jolla, CA, USA), and the significance was determined using ANOVA or Student’s t test. A p-value < 0.05 was considered statistically significant.

## Results

### HER3 Is Differentially Expressed in Basal and Claudin Type TNBC Cells

**Figure 1:**
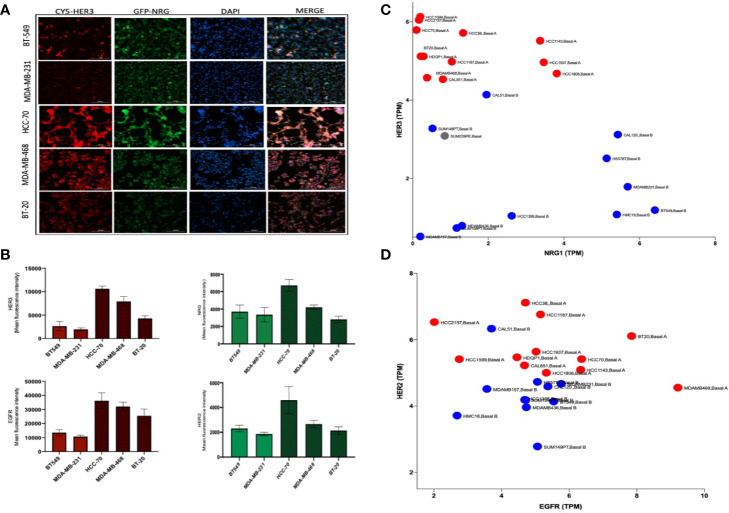
HER3 differentiates Basal from Claudin type Triple Negative Breast Cancer (TNBC). **(A)** Immunofluorescence analysis of HER3 and NRG expression in Basal (HCC-70, BT-20, and MDA-MB-468) and Claudin type (MDA-MB-231 and BT549) TNBC showing a higher expression of HER3 in the Basal compared to the Claudin subtype and variable Neuregulin expression. **(B)** Immunofluorescent quantification of EGFR, HER2, HER3, and NRG in the cell lines shown in **(A)**. Gene expression profile in a panel of Basal (Basal A -shown in red) and Claudin type (Basal B – shown in blue) TNBC cell lines using the online database DepMap showing transcripts per million (TMP) of HER3 and NRG **(C)** and EGFR and HER2 **(D)**.

NRG and HER3 have been shown to be differentially expressed in different TNBC cancer lines (21). Of the cell lines used in this study three are classified as Basal type, BT-20, MDA-MB-468, HCC-70 and two are characterized as Claudin type, MDA-MB-231 and BT-549, TNBC. Immunofluorescence analysis of the Basal and Claudin type TNBC cells confirmed the predominant expression of HER3 in the Basal subtype compared to the Claudin type cell lines which displayed a higher expression of NRG but not the HER3 receptor (**Figures 1A, B**), as previously reported by Momeny et al., (21). Furthermore, we show a similar pattern in EGFR and HER2 expression between Basal and Claudin type TNBC (**Figure 1B**). To further validate these findings, we analyzed their expression levels using the online repository (https://depmap.org/portal/) and found an inverse relationship between the expression of NRG and HER3 between these subtypes of TNBC (**Figure 1C**). EGFR and HER2 also show a discrete differentiation between the two subtypes as shown in **Figure 1D**. Please note that Basal and Claudin type TNBC can also be referred to as Basal A and Basal B TNBC, resembling basal like or claudin low tumours, respectively (22, 23). For ease of identification of the cell lines in the online database we have kept the annotation provided in the repository in **Figures 1C, D**.

### Differential Inhibitor-Induced HER3 Compensation Between Basal and Claudin Subtypes of TNBC

**Figure 2:**
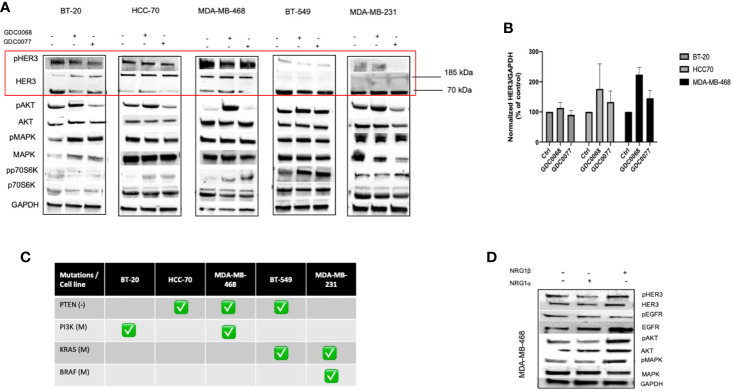
Differential HER3 response to AKT and PI3K inhibitors in Basal and Claudin type TNBC. **(A)** Biochemical assessment of the effect of AKT (GDC0068) and PI3K (GDC0077) inhibition in a panel of Basal and Claudin type Triple Negative Breast Cancer (TNBC) showing expression and upregulation of total and phosphorylated HER3 and downstream signaling in the Basal type TNBC cells (BT-20, HCC70, and MDA-MB-468). Cells were treated for 48h with the respective kinase inhibitors at 1uM **(B)** HER3 quantification in the TNBC cell lines tested in A and their corresponding mutational status of PTEN, PI3K, KRAS, and BRAF **(C)**. **(D)** Biochemical signalling analysis in response to NRG- 1α and -1β in MDA-MB-468 showing increased signalling with NRG- 1β isoform.

HER3 upregulation has been shown to be a feedback mechanism responsible for resistance to drug inhibition in breast cancer (24–26). We investigated the effect of PI3K (GDC0077) and AKT (GDC0068) inhibition on the level of HER3 and downstream signaling (**Figures 2A, B**). We found increased HER3 expression and downstream signaling in the Basal but not Claudin type TNBC cell lines, demonstrating a differential response to these drugs in these TNBC subtypes. In the Basal type TNBC cell lines, the extent of HER3 upregulation was cell line dependent. The AKT inhibitor, GDC0068 showed the highest levels of HER3 upregulation, irrespective of PTEN or PI3K status. The mutational status of the cell lines tested is shown in **Figure 2C**. Phosphorylation of HER3 and downstream signal transduction was apparent in all Basal TNBC cell lines, demonstrating an active involvement of HER3 signaling in Basal type TNBC. In contrast, the Claudin type TNBC cell lines displayed little to no phosphorylated or total membrane HER3. The 70 kDa soluble HER3 isoform was found in all cells but its role remains to be elucidated. The apparent increase in phosphorylated AKT when treated with the GDC0068 inhibitor is caused by the drug itself which inhibits the dephosphorylation of AKT and has previously been reported in other studies (24, 27, 28).

In accordance with previous studies, we show that NRG-1α induced phosphorylation of HER3 but to a smaller degree than NRG-1β (**Figure 2D**). Interestingly, the NRG-1β isoform also increased the expression but not phosphorylation of EGFR to a greater extent than the NRG-1α isoforms. These result indicate that signaling in the presence of NRG-1β is aided by the recruitment of EGFR, followed by heterodimerization with HER3 and its subsequent phosphorylation. Furthermore, cells treated with NRG-1β showed no morphological changes (**Figure S1**) indicating a predominant effect in cell survival rather than having a role mediating invasion and metastasis.

### Microenvironmental Neuregulin Reduces Efficacy to Small Molecule Inhibitors in Basal Like TNBC Cells

**Figure 3:**
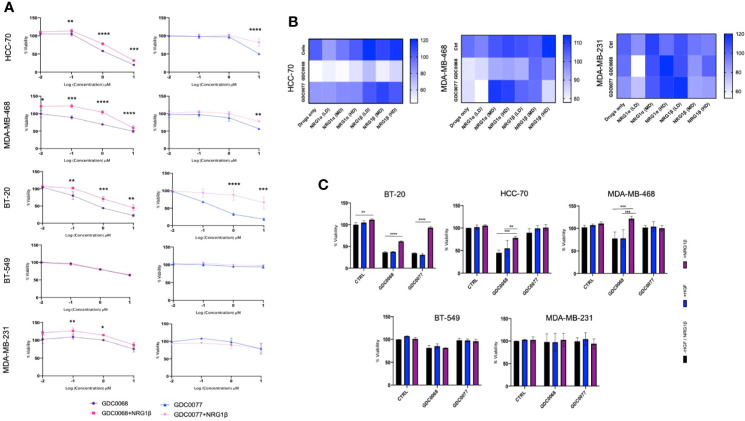
Neuregulin but not Hepatocyte Growth Factor decreases sensitivity to kinase inhibitors in Basal type Triple Negative Breast Cancer (TNBC). **(A)** Viability assay assessing the effect of Neuregulin-1β (50 ng/ml) in the presence of the small molecule inhibitors GDC0068 (1uM) and GDC0077 (1uM) after 96h treatment in a panel of TNBC cell lines in BT-20, HCC-70, MDA-MB-468, BT-549, and MDA-MB-231 and **(B)** Dose dependent effects of NRG- 1α and -1β isoforms [Low Dose (LD) 5 ng/ml, Medium Dose (MD) 50 ng/ml, and high dose (HD) 100 ng/ml] in the presence or absence of GDC0068 (1uM) or GDC0077 (1uM) for 96h in HCC-70, MDA-MB-468, and MDA-MB-231 TNBC. **(C)** Effect of HGF treatment (50 ng/ml) after 96h in the presence or absence of GDC0068/GDC0077 at 1uM. Drug response graphs and Heat map graphs show CellTiter-Glo luminescence viability measurements at the end of the experiments compared to untreated control. Experiments were performed in triplicate. Data are means ± SD]. Drug response graphs were analyzed using two-way analysis of variance (ANOVA)/Tukey’s multiple comparison test, *<0.05, **P < 0.01, ***P < 0.001, ****P < 0.0001].

Neuregulin has been implicated as a potent microenvironmental growth factor mediating resistance in different cancers including breast cancer. We next investigated the effect of a neuregulin rich microenvironmental on Basal and Claudin type TNBC. The cell lines tested in this study, are known to display different cytotoxic profiles to the AKT inhibitor GDC0068 or the PI3K inhibitor GDC0077. To determine if NRG decreases sensitivity to these drugs, cells were treated with increasing concentrations of the AKT inhibitor GDC0068 or the PI3K inhibitor, GDC0077, alone or in the presence of the NRG-1β. In the presence of NRG-1β, the drugs showed a significantly reduced efficacy at the doses tested (**Figure 3A**). At the treatment dose (1uM) the presence of NRG-1β, decreased sensitivity to GDC0068 by approximately 20% and 40% in the Basal cell lines BT-20, HCC-70 and MDA-MB-468. The PI3K inhibitor showed an increase in viability in the presence of NRG-1β of between 10% -20% in the HCC-70 and MDA-MB-468 and up to 55% in BT-20 cell line. In the Claudin type cell line, MDA-MB-231 and BT-549, which express the lowest levels of HER3, NRG-1β reduced viability in the presence of the GDC0068 inhibitor but not the PI3K inhibitor, albeit at lower levels than the high HER3 expressing cells. Together, these results demonstrate that NRG-1β reduces sensitivity to PI3K/AKT inhibitors in TNBC cancer cells, specifically in the Basal subtype.

NRG isoforms are known to display different affinities for the HER3 receptor, conferring different activation potential. To determine the effect of different NRG isoforms in TNBC, NRG -1α, and 1β isoforms were tested. We assessed cell viability after 96 h at different doses of the isoforms in the presence or absence of the small molecule inhibitors, GDC0068 and GDC0077 (**Figure 3B**). We show that NRG isoforms exhibit differential proliferative effects in the TNBC cell lines tested. The Basal subtype HCC-70 and MDA-MB-468 cells had a more uniform response to different doses and NRG isoforms showing a dose dependent increase in cell viability with medium (50 ng/ml) and high doses (100 ng/ml) of NRG-1α and -1β isoforms. Low doses of NRG isoforms in the absence of any drugs showed mixed results in the cell lines tests.

Lastly, we tested the effect of GDC0068 and GDC0077 in the presence of Hepatocyte Growth Factor (HGF) since the cell lines tested also express cMET (29). Interestingly, we found that HGF does not mediate a significant increase in viability in these cell lines (**Figure 3C**), highlighting the role of HER3 and NRG signaling in response to the small molecule inhibitors tested.

### Dual Targeting With EGFR Inhibitor and PI3K/AKT Inhibitors Reduces NRG-HER3 Mediated Resistance in TNBC

**Figure 4:**
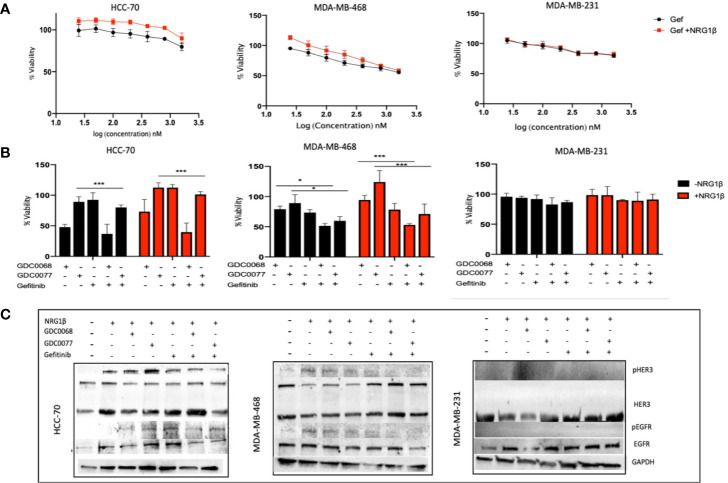
Gefitinib in combination with AKT/PI3K inhibitors sensitizes Basal type Triple Negative Breast Cancer (TNBC). **(A)** HCC-70, MDA-MB-468, and MDA-MB-231 TNBC cells treated in the presence of NRG-1β (50 ng/ml) does not significantly decrease sensitivity to gefitinib (200 nM) after 96 h. **(B)** Viability assay of TNBC cells as in **(A)** treated with the small molecule inhibitors GDC0068 (1uM), GDC0077 (1uM), gefitinib (200 nM), or a combination of the drugs +/- NRG1β (50 ng/ml) for 96h. In the HCC-70 and MDA-MB-468 cell lines, combination therapy demonstrated decreased viability compared to the MDA-MB-231 cell line which showed no significant difference in response, regardless of treatment **(C)** Biochemical analysis of the p/EGFR and p/HER3 showing decreased but not abolished phosphorylated proteins indicating a potent and sustained signaling in presence of NRG1β in the Basal type TNBC cells, HCC-70, and MDA-MB-231; Cells were treated for 48h at the drug concentrations shown in **(B)**. The Claudin type MDA-MB-231 cell line showed no HER3 phosphorylation. Drug response graphs show CellTiter-Glo luminescence viability measurements at the end of the experiments compared to untreated control and analyzed using two-way analysis of variance (ANOVA)/Tukey’s multiple comparison test, *<0.05, ***P<0.001]. Experiments were performed in triplicate. Data are means ± SD].

Studies have shown that EGFR and HER3 upregulation and activation mediate resistance to small molecule inhibitors (30). We next tested the effect of NRG-1β in the presence of dual targeting with the EGFR inhibitor, gefitinib, in combination with GDC0068 or GDC0077. Tao et al. (24) has previously demonstrated improved response rates with dual inhibition of EGFR/HER3. **Figure 4A** shows that NRG-1β does not significantly reduce the efficacy of gefitinib demonstrating drug dependent responses in the presence of NRG. However, combination therapy showed cell line specific responses probably linked to the expression of HER family members and/or mutational fingerprint (**Figure 4B**). Combination therapy was able to reduce viability both in the absence or presence of NRG-1β in the microenvironment in the Basal TNBC cell lines but not in the Claudin type cell lines. Furthermore, combination therapy showed decreased phosphorylation of EGFR and HER3 and downstream signaling in the presence of NRG-1β in the two Basal cell lines but not the Claudin type cell line (**Figure 4C**). In the MDA-MB-231 Claudin type cell line, the combination of gefitinib and AKT/PI3K inhibition had no effect with continued phosphorylation of MAPK (data not shown), confirming a different reliance on EGFR family members and dominant signaling pathway. These results demonstrate differential wiring mechanisms in the two subtypes of TNBC, which should be addressed when considering the therapeutic strategy.

Interestingly, Wilson et al. (31) demonstrated sensitivity, in a subset of non-amplified HER2 cancers, to the dual EGFR/HER2 inhibitor Lapatinib, effect attributed to NRG secreting cells. We found that TNBC cell lines are not sensitive to Lapatinib as a single agent (**Figure S2**). However, the combination of lapatinib and the GDC0068 or GDC0077 was superior to single agent treatment in the presence or absence of NRG-1β (**Figure S3**). The superior cytotoxicity to the combination of drugs was seen in the Basal but not in the Claudin TNBC subtype, confirming different pathway reliance in the two TNBC subtypes. Whether the effect of this combination is due to GDC0068 or GDC0077 dependent upregulation of HER3 or due to the additive effects of the drugs remains to be elucidated. Thus, NRG produces a favorable microenvironment for cancer cell survival through activation of the PI3K/AKT pathway and forms an actionable mechanism of drug resistance in Basal type TNBC.

Cumulatively, these results show that resistance mechanisms to targeted single agent treatments triggers feedback mechanisms through the upregulation and phosphorylation of EGFR and HER3 in the Basal but not Claudin TNBC subtype. Interestingly, EGFR inhibition with the small molecule inhibitor gefitinib results in the upregulation and phosphorylation of HER3 demonstrating the promiscuous nature of the EGFR-HER3 dimer and its role in mediating resistance in TNBC.

### Complete Inhibition of the Dominant Pathway Reverses NRG/EGFR/HER3 Mediated Resistance in TNBC

**Figure 5:**
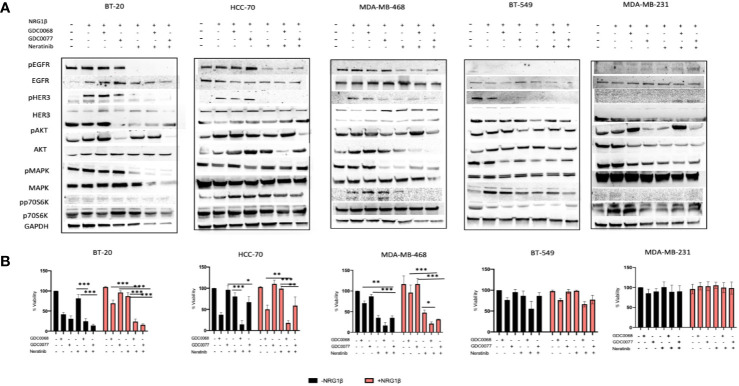
Pan HER targeting sensitizes Basal type but not Claudin type Triple Negative Breast Cancer (TNBC) cells to AKT and PI3K inhibition. **(A)** Biochemical assessment of downstream signaling in the PI3K/AKT signaling pathway after combination therapy with neratinib (300 nM) and GDC0068 (1 uM) or GDC0077 (1 uM) after 48 h, showing deceased signaling in the Basal TNBC cell lines but not in the Claudin type TNBC. **(B)** Viability assay of TNBC cells treated with the small molecule inhibitors GDC0068, GDC0077, neratinib, or a combination of the drugs for 96h [drug treatments as in **(A)**]. In the BT-20, HCC-70, and MDA-MB-468 cell lines, combination therapy demonstrated decreased viability compared to the MDA-MB-231and BT-549 cell line which showed no added benefit of combination therapy. Drug response graphs show CellTiter-Glo luminescence viability measurements at the end of the experiments compared to untreated control and analyzed using two-way analysis of variance (ANOVA)/Tukey’s multiple comparison test, *<0.05, **P < 0.01, ***P<0.001]. Experiments were performed in triplicate. Data are means ± SD].

We hypothesized that a pan EGFR inhibitor may show superior cytotoxic effects in TNBC cell lines that rely on EGFR signaling. We used the FDA approved irreversible pan HER inhibitor, neratinib, and biochemically analyzed the effect of combination therapy with the GDC0068 and GDC0077 inhibitors. We found that Basal but not Claudin type TNBC cells treated with neratinib showed decreased signaling when combined with the AKT or PI3K inhibitor (**Figure 5A**). In the Claudin type TNBC, neratinib showed no synergism to the AKT/PI3K inhibitor. Combination therapy showed superior cytotoxic efficacy in all Basal type TNBC cells in the presence or absence of high levels of NRG (**Figure 5B**). This is particularly important in metastatic cancer where cells exposed to high levels of NRG are likely to decrease sensitivity to these drugs and may contribute to resistance in these locations. Furthermore, it has been previously shown that neuregulin mediates resistance to Pi3K inhibitors through the mTORC1 pathway (32). Unsurprisingly, the cytotoxic effect was also related to the mutational status of the cells. Thus, Basal type TNBC cells with PTEN mutations will show superior responses when treated with an AKT inhibitor, compared to the PI3K inhibitor, in combination with complete inhibition of the HER family of receptors to achieve complete inhibition of the dominant signaling pathway.

Lastly, we also tested the experimental pan inhibitor sapitinib, a reversible ATP inhibitor, to determine if a similar pattern is seen, in the Basal and Claudin type TNBC cell lines. We found that NRG-1β does not significantly decrease sensitivity to sapitinib (data not shown). Furthermore, we found increased sensitivity to dual inhibition with sapitinib or the AKT/PI3K inhibitor in the HCC-70 and MDA-MB-468 cell lines more effectively compared the MDA-MB-231 cell line (**Figure S3A**). While, combination therapy with sapitinib reduced phosphorylation of ERK we did not find a significant decrease in the ki67 proliferation marker as shown in the ki67 stained images (**Figure S3C, D**).

Cumulatively, these results show the inherent versatility of TNBCs to restore signaling after single pathway inhibition whether at the level of the receptor or a downstream signaling target.

Specifically, a subset of TNBC cells, preferentially employ the EGFR-HER3-AKT pathway. In these cells, complete abrogation of the dominant signaling network through simultaneous therapeutic targeting confers survival vulnerability in TNBC. These results demonstrate differential mechanisms of resistance and indicate that EGFR expression alone is not a reliable indicator of sensitivity to treatments. Given the important role of the EGFR and HER3 heterodimer in driving TNBC growth, HER3 expression may be used to identify patients who would respond to targeted inhibition of the EGFR/HER/AKT pathway from those who would benefit least from such an approach.

## Discussion

TNBC remains one of the hardest subtypes of BC to treat. This resilience can, at least in part, be explained by the lack of classical oncogene addiction, making TNBC less vulnerable to therapies that target a single protein or pathway. In this study, we show differential dependencies in the NRG/HER3/AKT axis inherent within TNBC resistance networks, that can be disrupted through complete abrogation of the HER receptor family and the PI3K/AKT signaling axis. We show that TNBC cells of the Basal subtype express the HER3 protein but not the Claudin subtype. Furthermore, we show that inhibition of the PI3K/AKT pathway results in the upregulation of the HER3 receptor itself contributing to resistance to these therapies. Upregulation of the HER3 receptor triggers a switch toward the PI3K/AKT signaling pathway which can be further maintained by the presence of the autocrine or paracrine growth factor, NRG. To our knowledge, the importance of NRG has not been studied in the context of PI3K/AKT inhibition in TNBC. We believe that the results presented provide a rationale for targeted therapy in TNBC and that the microenvironment in which TNBC resides plays a significant role in drug efficacy.

NRG showed differential effects in cell viability in the cell lines tested which was shown to be isoform and dose dependent. At low doses NRG-1α caused an apparent decrease in cell viability but not at high doses. Overall, NRG-1β showed a more potent effect in increasing cell viability compared to the NRG-1α isoform. Moreover, NRG-1β decreased sensitivity to the AKT and PI3K inhibitors in a cell type specific manner. The complexity of the effects of NRG have been confirmed in numerous other studies including Watson et al. (33) and Eckert et al. (34). Furthermore, we found that NRG on its own displays cell type, dose concentration and dose frequency (data not shown) dependent effects possibly related to the level of HER3 expression and the frequency of receptor internalization, corroborating multiple reports on the complexity of NRG isoforms and its numerous cellular effects.

NRG has been strongly implicated in treatment failure in HER2+ BC and numerous other cancers. Furthermore, since NRG can cause autocrine, paracrine and juxtacrine signaling its effect can significantly affect therapeutic intervention. This is best illustrated in certain cancers where targeted therapy is effective at controlling metastasis but does not provide effective control for brain metastasis (18). Previous studies have shown that BC which express HER3 are associated to higher rate of brain metastasis, while induction of HER3 is associated with the development of brain metastasis from both breast and lung cancers (21, 35–37). Furthermore, Kodack et al. (18) demonstrated that brain metastasis in the HER2 amplified setting showed increased phosphorylation of EGFR and HER3. Our study shows a strong maintenance of increased phosphorylation of EGFR and HER3 and downstream signaling in the presence of Neureuglin-1β which was unable to be abolished with the competitive HER3 antibody in combination with GDC0068 or GDC0077 (data not shown). Complete inhibition of the HER receptor family and of the PI3K or AKT node was necessary to achieve complete inhibition of the pathway. The fact that the MDA-MB-231 does not respond to this approach, highlights a differential pathway dependence.

Cumulatively, our findings suggest that autocrine and paracrine NRG signaling can significantly decrease sensitivity and therefore efficiency to targeted inhibition. This is an important consideration as differential effects may be seen in cases where the cancer cells themselves do not express NRG but the therapeutic response can be reduced if NRG is present in the microenvironment.

The therapeutic approach used in this study may be beneficial for both *in situ* and metastatic TNBC as well as in other cancers that show preferential NRG/HER3/AKT signaling for growth or survival. Although the clinical relevance of these studies needs to be further investigated in animal studies and human patient cohorts, the findings suggest that TNBC cancer cells display differential resistance responses to microenvironmental factors thus reflecting fundamental differences in signaling reliance between Basal and Claudin type TNBC.

In conclusion, the results shown herein provide a mechanistic basis for NRG dependent and independent therapeutic resistance in TNBC and identify translatable treatment strategies for the Basal subtype of TNBC. NRG mediated HER3 signaling is actively used by TNBC cells and thus targeted therapy to inhibit its effect may delay the onset of metastasis in microenvironments such as the brain where NRG is often expressed at higher levels. Thus, identifying cells which show a reliance on HER members may be used to stratify those cancers which will respond to targeted therapy of the HER-PI3K-AKT axis.

## Data Availability Statement

The datasets presented in this study can be found in online repositories. The names of the repository/repositories and accession number(s) can be found in the article/**Supplementary Material**.

## Author Contributions

Experimental conception and design was performed by NS, PH, and UM. Western blot experiments were performed and analyzed by NS, BA, KL, VZ, and LG. Cell viability and microscopy experiments were performed and analyzed by NS and LG. Manuscript drafting performed by NS. Manuscript editing performed by NS, BA, KL, VZ, PH, and UM. All authors contributed to the article and approved the submitted version.

## Funding

Funding for conduct of the study is provided by the NIH R01-CA211223 and ESSCO-MGH Breast Cancer Research Fund 230334. The funding bodies had no role in design, data collection or analysis, interpretation, or writing of the manuscript.

## Conflict of Interest

The authors declare that the research was conducted in the absence of any commercial or financial relationships that could be construed as a potential conflict of interest.

## Supplementary Material

Supplementary Figure 1 Treatment of Basal type TNBC cell lines with exogenous HRG does not alters cell morphology. Serum-starved cells were treated with Neuregulin1β alone in the presence of GDC0068 or GDC0077 for 48 h then imaged by light microscopy (images acquired at 10x magnification).

Supplementary Figure 2 Combination therapy with lapatinib decreases sensitivity in Basal type TNBC. Viability assay assessing the effect of combination therapy between the small molecule inhibitors GDC0068 (1uM) and GDC0077 (1uM) and lapatinib (200 nM) in the presence of absence of NRG-1β (50 ng/ml) after 96h. Drug response graphs show CellTiter-Glo luminescence viability measurements at the end of the experiments compared to untreated control.

Supplementary Figure 3 Combination treatment with sapitinib decreases viability in Basal but not Claudin type TNBC cells. **(A)** Viability assay assessing the effect of combination therapy with the pan inhibitor, sapitinib (0.5 uM) and the AKT inhibitor GDC0068 or the Pi3K inhibitor GDC0077 +/-Neuregulin-1β (50 ng/ml) after 96h in HCC-70, MDA-MB-468 and MDA-MB-231. **(B)** Biochemical assessment of downstream signaling in the PI3K/AKT signaling pathway after combination therapy with sapitinib and GDC0068 or GDC0077 in MDA-MB-468. **(C)** Immunofluorescence staining of the proliferation marker Ki67 showing reduced cell proliferation with pan HER family inhibition and the GDC0068 or GDC0077 tyrosine kinase inhibitors and **(D)** Mean Fluorescence Intensity of Ki67 proliferation marker analyzed using Biotek Cytation5. Viability graphs show CellTiter-Glo luminescence measurements at the end of the experiments compared to untreated control and analyzed using the two-way analysis of variance (ANOVA)/Tukey’s multiple comparison test, *<0.05, **P < 0.01, ***P<0.001, ****P < 0.0001]. Experiments were performed in triplicate. Data are means ± SD].
