# Supplementary figures and images for: HER3 Differentiates Basal From Claudin Type Triple Negative Breast Cancer and Contributes to Drug and Microenvironmental Induced Resistance
Source: Front Oncol. 2020 Nov 20;10:554704. doi: 10.3389/fonc.2020.554704 (PMC7715030; doi:10.3389/fonc.2020.554704)

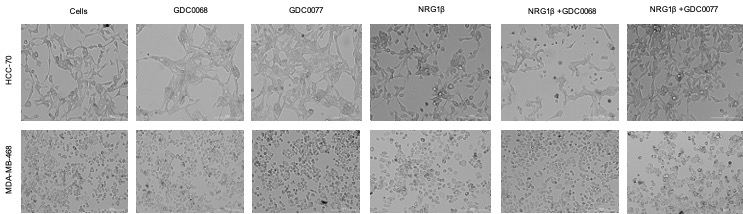

Supplement: Supplementary Figure 1 — Treatment of Basal type TNBC cell lines with exogenous HRG does not alters cell morphology. Serum-starved cells were treated with Neuregulin1β alone in the presence of GDC0068 or GDC0077 for 48 h then imaged by light microscopy (images acquired at 10x magnification). [file Image_1.jpeg]

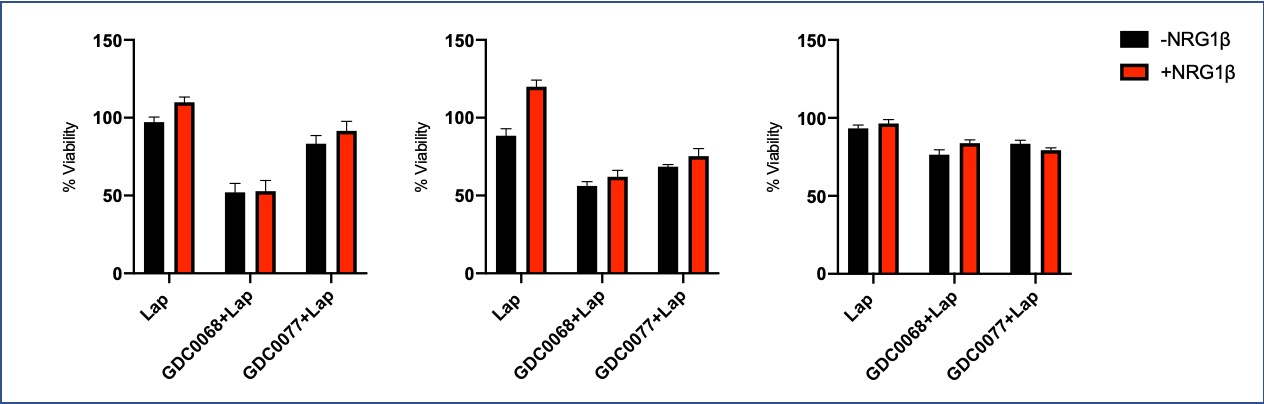

Supplement: Supplementary Figure 2 — Combination therapy with lapatinib decreases sensitivity in Basal type TNBC. Viability assay assessing the effect of combination therapy between the small molecule inhibitors GDC0068 (1uM) and GDC0077 (1uM) and lapatinib (200 nM) in the presence of absence of NRG-1β (50 ng/ml) after 96h. Drug response graphs show CellTiter-Glo luminescence viability measurements at the end of the experiments compared to untreated control. [file Image_2.jpeg]

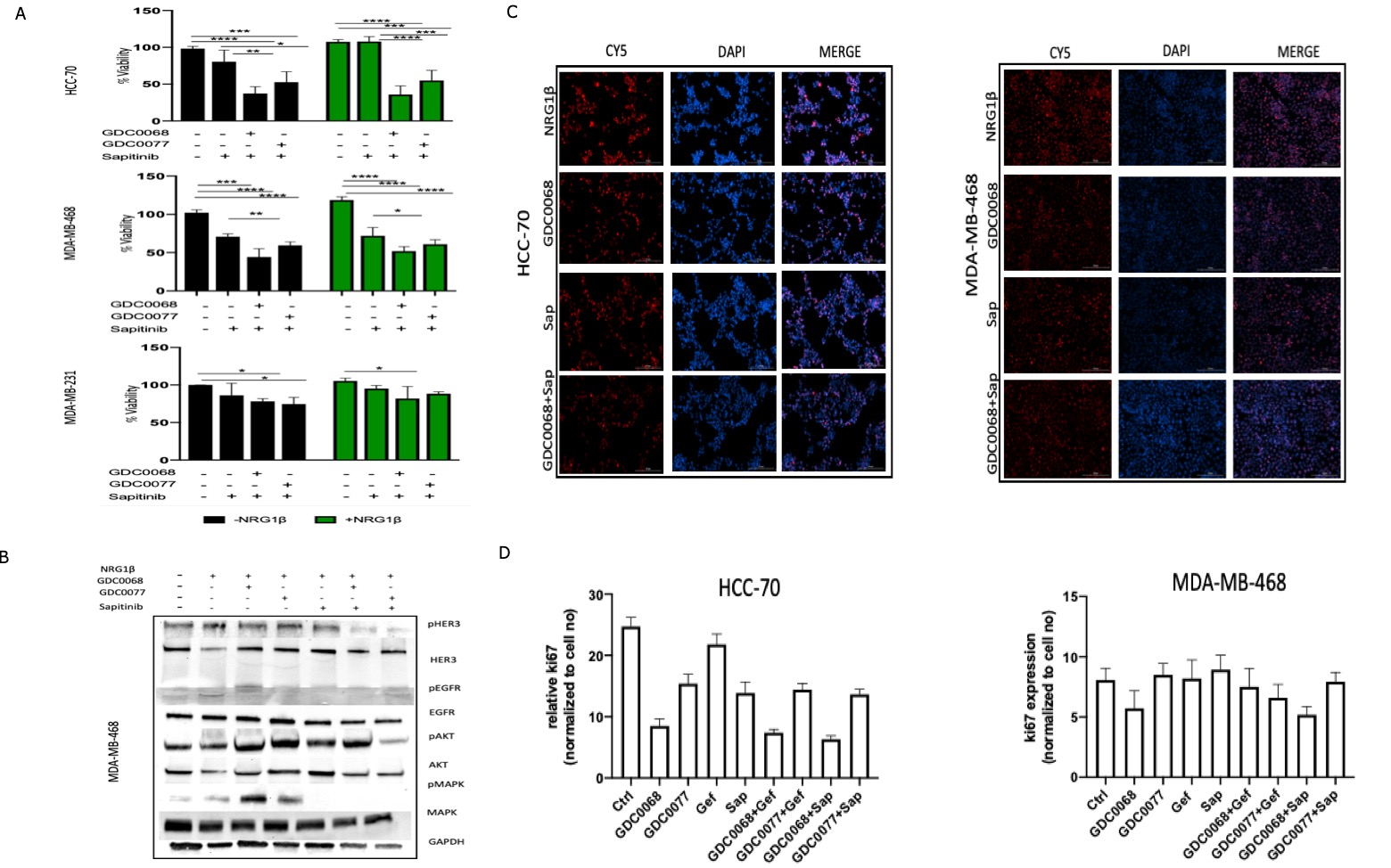

Supplement: Supplementary Figure 3 — Combination treatment with sapitinib decreases viability in Basal but not Claudin type TNBC cells. (A) Viability assay assessing the effect of combination therapy with the pan inhibitor, sapitinib (0.5 uM) and the AKT inhibitor GDC0068 or the Pi3K inhibitor GDC0077 +/-Neuregulin-1β (50 ng/ml) after 96h in HCC-70, MDA-MB-468 and MDA-MB-231. (B) Biochemical assessment of downstream signaling in the PI3K/AKT signaling pathway after combination therapy with sapitinib and GDC0068 or GDC0077 in MDA-MB-468. (C) Immunofluorescence staining of the proliferation marker Ki67 showing reduced cell proliferation with pan HER family inhibition and the GDC0068 or GDC0077 tyrosine kinase inhibitors and (D) Mean Fluorescence Intensity of Ki67 proliferation marker analyzed using Biotek Cytation5. Viability graphs show CellTiter-Glo luminescence measurements at the end of the experiments compared to untreated control and analyzed using the two-way analysis of variance (ANOVA)/Tukey’s multiple comparison test, *<0.05, **P < 0.01, ***P<0.001, ****P < 0.0001]. Experiments were performed in triplicate. Data are means ± SD]. [file Image_3.jpeg]
